# Brazilian Table Olives: A Source of Lactic Acid Bacteria with Antimycotoxigenic and Antifungal Activity

**DOI:** 10.3390/toxins15010071

**Published:** 2023-01-13

**Authors:** Luara Simões, Natália Fernandes, José Teixeira, Luís Abrunhosa, Disney Ribeiro Dias

**Affiliations:** 1 Biology Department, Federal University of Lavras, Lavras 37200-900, Brazil; 2 Centre of Molecular and Environmental Biology, University of Minho, 4710-057 Braga, Portugal; 3 Chemistry Department, University of California, Davis, CA 95616, USA; 4 CEB—Centre of Biological Engineering, University of Minho, 4710-057 Braga, Portugal; 5 LABBELS—Associate Laboratory, Braga/Guimarães, Portugal; 6 Department of Food Science, Federal University of Lavras, Lavras 37200-900, Brazil

**Keywords:** Aflatoxins, antifungal compounds, biocontrol, lactic acid bacteria, mycotoxins

## Abstract

Food and feed contamination by fungi, especially by toxigenic ones, is a global concern because it can pose serious health problems when the production of mycotoxins is involved. Lactic acid bacteria (LAB), well-known for fermenting foods, have been gaining attention for their antifungal and anti-mycotoxin properties. This work tested 14 LAB strains isolated from naturally fermented Brazilian table olives for growth inhibition of *Aspergillus flavus*, *Aspergillus carbonarius*, *Penicillium nordicum*, and *Penicillium expansum*. The strains *Lacticaseibacillus paracasei* subsp. *paracasei* CCMA 1764, *Levilactobacillus brevis* CCMA 1762, and *Lactiplantibacillus pentosus* CCMA 1768 showed the strongest antifungal activity, being more active against *P. expansum*. Aflatoxin B1 (AFB1), ochratoxin A (OTA), and patulin (PAT) production was reduced essentially by mycelia growth inhibition. The main organic acids detected in the cell free supernatant (CFS) were lactic and acetic acids. Tested LAB exhibited adsorption capacity against AFB1 (48–51%), OTA (28–33%), and PAT (23–24%). AFB1 was converted into aflatoxin B2a (AFB2a) by lactic and acetic acids produced by the strain CCMA 1764. A similar conversion was observed in solutions of these organic acids (0.1 M). These findings demonstrate the potential of isolated LAB strains as natural agents to control toxigenic fungi and their mycotoxins in fermented products, such as table olives.

## 1. Introduction

Phytopathogenic fungi are responsible for major socioeconomic impacts, undermine food safety, and consequently affect public health [1]. In addition to causing detrimental sensory changes in food’s taste, odor, color, and texture, fungi are also responsible for the chemical deterioration of foods, especially when they produce mycotoxins [2]. This problem is a global menace, as approximately 20% of worldwide commodities are contaminated with mycotoxins levels above the lower legal limits, and up to 60–80% might contain detectable levels of at least one mycotoxin [3].

Aflatoxins (AFLs) are one of the best-known groups of mycotoxins, being found mainly in tropical and subtropical regions associated with commodities, such as cotton, peanuts, spices, pistachios, and corn [4]. *Aspergillus flavus* and *Aspergillus parasiticus* are the main sources of aflatoxins [5]. Nonetheless, other mycotoxins, such as ochratoxin A (OTA) and patulin (PAT), are also potentially dangerous in products, such as cereals, coffee, wines, or apples and derived foodstuffs [6]. OTA can be produced by several species of the *Aspergillus* and *Penicillium* genera, among which *A. carbonarius*, *A. ochraceus*, *P. verrucosum,* and *P. nordicum* are the most well-recognized [7]. On the other hand, PAT is mainly produced by *Penicillium* species, with *P. expansum* being the most important producer [8]. Interestingly, some of these species can proliferate in salt-rich environments [9], and species, such as *A. flavus*, *A. ochraceus* [10], *P. expansum* [11], and *P. verrucosum* [12], have been isolated from table olives or from the respective fermentation brine surface.

In recent years, some studies have shown the inhibitory activity of LAB against fungi and mycotoxins. For example, the bacterium *Lactiplantibacillus plantarum* [13] and strains of *Lactiplantibacillus pentosus* and *Lacticaseibacillus paracasei* subsp. *paracasei* [14] showed antifungal properties against *P. nordicum*. The bacterium *Leuconostoc mesenteroides* subsp. *mesenteroides* LB7 inhibited the growth of *P. expansum* and reduced patulin in commercial fruit juices [15], and the strain *Levilactobacillus brevis* 8-2B inhibited the growth of *A. carbonarius* and ochratoxin A production [16]. LAB, like many probiotics, are *generally recognized as safe* (GRAS) [17]. During fermentation, LAB produce acidic substances that reduce the pH value of the environment and cause intracellular acid stress, resulting in unfavorable conditions for the growth of fungi in food products [18]. These acidic substances, such as lactic acid (LA), acetic acid (AA), propionic acid, formic acid, phenyllactic acid, and fatty acids, have shown antifungal properties [13,19,20]. Table olives are also susceptible to toxigenic fungi growth, either in the field or during their processing once it usually involves a fermentation step that takes several months [11,21,22,23]. In addition, some studies reported the contamination of table olives by aflatoxin B1 and ochratoxin A [24,25,26], and patulin [11]. Thus, the presence of toxigenic fungi or mycotoxins in table olives raises concerns about the safety of this product and demands strategies to efficiently avoid the development of toxic fungi during their processing.

Considering the growing interest in bio-strategies to replace synthetic chemical preservatives, the impact of toxigenic fungi and mycotoxins in table olives on human health, and the potential of LAB for food preservation, this study aims at investigating the antifungal and antimycotoxigenic properties of LAB isolated from naturally fermented Brazilian table olives. Therefore, growth inhibition studies were conducted using *A. flavus*, *A. carbonarius*, *P. nordicum,* and *P. expansum,* and the respective production of their mycotoxins, aflatoxin B1 (AFB1), ochratoxin A, and patulin was monitored over the assays. In addition, some LAB strains were further characterized for their mycotoxin biotransformation and binding activity.

## 2. Results

### 2.1. Screening of LAB for Antifungal Properties

**Figure 1:**
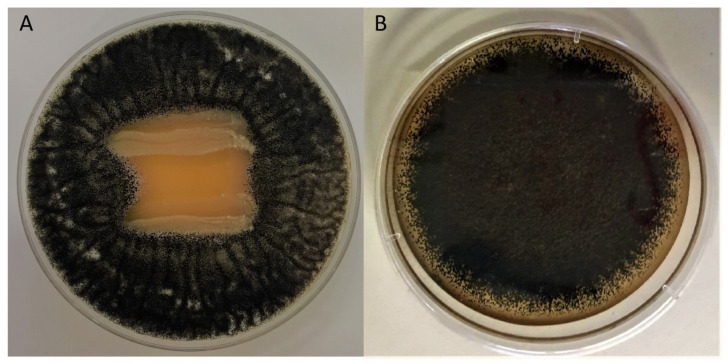
Example of the antifungal activity against *A. carbonarius* 01UAs293 determined by the “overlay method”. (**A**) *L. brevis* CCMA 1762 (inhibition halo), (**B**) *L. paracasei* CCMA 1770 (no effect).

Fourteen LAB (Table S1) were tested by “dual-culture overlay assay” against the fungi *A. flavus* MUM 08.201, *A. carbonarius* 01UAs293, *P. nordicum* MUM 08.16, and *P. expansum* MUM 17.86. These LAB strains were isolated from naturally fermented Brazilian table olives, an environment considered extreme for these bacteria, as it contains high levels of polyphenols and sodium chloride [27]. In general, most tested strains demonstrated some antifungal activity (Table 1), particularly against species of the *Penicillium* genus, which were more susceptible than fungi of the *Aspergillus* genus. Among the *Penicillium*, *P. nordicum* MUM 08.16 was particularly sensitive to many of the tested LAB strains and species (seven strains with +++, two strains with ++, and four strains with +). The strains *L. paracasei* CCMA 1763, *L. pentosus* CCMA 1768, *L. paracasei* CCMA 1775, *L. brevis* CCMA 1762, and *L. paracasei* CCMA 1764 showed the most potent antifungal activity against target fungal strains, with an inhibition halo around bacteria lines being formed as illustrated in Figure 1. From all strains tested, three isolates (*L. brevis* CCMA 1762, *L. paracasei* CCMA 1764, and *L. pentosus* CCMA 1768) were selected to conduct further experiments, as they were the most competent in inhibiting fungal growth.

### 2.2. Plate Inhibition Analysis of Cell-Free Supernatants

The CFS of selected LAB strains (*L. paracasei* CCMA 1764, *L. pentosus* CCMA 1768, and *L. brevis* CCMA 1762) was tested in plates against the four fungi at two different concentrations (Table 2). For the *Aspergillus* species, 300 µL/mL of CFS was insufficient to inhibit fungi growth once the hyphal radial growth remained nearly the same as negative controls (without adding CFS). However, with the increase of CFS concentration to 500 µL/mL, the diameter of *A. carbonarius* and *A. flavus* decreased. An inhibition of approximately 100% was obtained with *L. paracasei* CCMA 1764 and *L. pentosus* CCMA 1768, while the CFS of bacteria *L. brevis* CCMA 1762 was less effective in inhibiting the growth of these fungi. In this case, a reduction of 40% in the growth of *A. carbonarius* and 78% in *A. flavus* was registered compared to controls.

Regarding the *Penicillium* species, both concentrations (300 and 500 µL/mL) of tested CFS affected fungal growth, but the effect was stronger with the higher dose. For *P. nordicum,* 300 µL/mL of CFS reduced the growth of the fungus between 37% to 41%, while with 500 µL/mL of CFS, all LAB could entirely suppress the radial growth in plates. Results for *P. expansum* show even more pronounced growth inhibitions, ranging from 78% to 92% with 300 µL/mL of CFS and between 77% and 100% with 500 µL/mL. The bacterium *L. brevis* CCMA 1762 was less active against this fungus, while *L. paracasei* CCMA 1764 and *L. pentosus* CCMA 1768 controlled their growth totally (Table 2).

### 2.3. Microplate Inhibition Analysis of Cell-Free Supernatants

The antifungal activity of CFS was also tested on 96-well microplates by recording the optical density (OD_600nm_) of wells over the growth incubation period (Table 2). All target fungi had their growth substantially suppressed by 500 µL/mL of CFS from *L. paracasei* CCMA 1764 and *L. pentosus* CCMA 1768, with the inhibition percentages ranging from 98 to 100%. On the other hand, the CFS of *L. brevis* CCMA 1762 at 500 µL/mL concentration showed a lesser antifungal potency against *A. carbonarius* (30%) and *A. flavus* (73%). Moreover, *L. pentosus* CCMA 1768 at 300 µL/mL did not effectively reduce the growth of the Aspergillus species, achieving reductions at most of 27%. On the contrary, *P. expansum* was still the most sensitive of the tested fungi; its growth was reduced by 87–93% with the 300 µL/mL concentration and by 96–100% with 500 µL/mL (Table 2). All tested LAB CFS were strongly active against this fungus. *P. nordicum* was also very sensitive to LAB CFS, showing inhibition of 95–100%, except when CFS of *L. brevis* CCMA 1762 was used at 300 µL/mL (16% of inhibition).

### 2.4. Analyses of Mycotoxins in Inhibition Assays

The mycotoxins AFB1 (from *A. flavus* growth), OTA (from *A. carbonarius* growth), and PAT (from *P. expansum* growth) were extracted from plates and microplates after 7 days of fungal growth and quantified by HPLC (Table 3). In samples containing 500 µL/mL of CFS from *L. paracasei* CCMA 1764 and *L. pentosus* CCMA 1768, there was an absence of mycotoxins, which corresponds to a 100% reduction compared to control samples. These results agree with data from Table 2, as no substantial fungal growth was detected in those conditions. On the other hand, the CFS of *L. brevis* CCMA 1762 at 500 µL/mL could not fully inhibit the production of the mycotoxins, with reductions of 98% and 95% being observed for AFB1, of 81% and 65% for OTA, and 95% and 93% for PAT in microplates and plates, respectively.

In samples containing 300 µL/mL of CFS, there was some reduction in the concentration of AFB1 compared to the positive controls. The reduction ranged from 8% (*L. brevis* CCMA 1762) to 31% (*L. paracasei* CCMA 1764) for microplate tests and from 18% (*L. brevis* CCMA 1762) to 29% (*L. paracasei* CCMA 1764) for plate tests. With this CFS concentration, no reduction in OTA production was observed. On the contrary, PAT reductions were substantially higher, ranging from 95% to 100% for the microplate assays and from 89% to 98% for the plate assays.

### 2.5. Analysis of Cell-Free Supernatant

**Figure 2:**
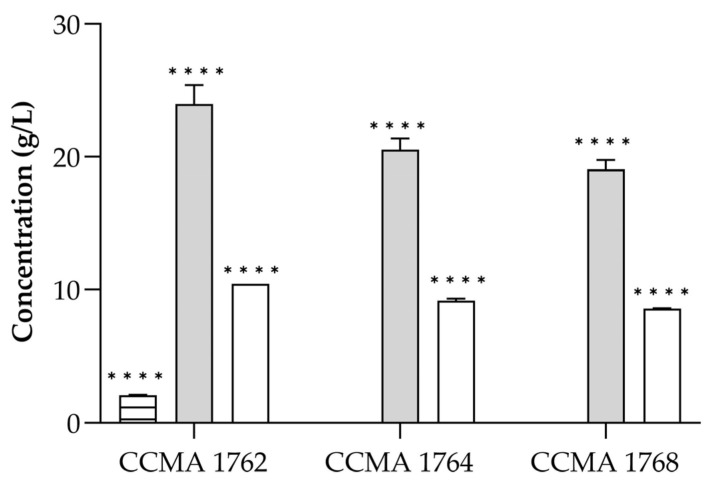
Concentration (g/L) of organic acids in LAB CFS. Lactic acid (grey), acetic acid (white), and citric acid (horizontal lines). The results are expressed as mean ± SD (*n* = 2). Statistical significance was determined by Tukey’s multiple comparisons test (**** *p* < 0.0001).

After confirming the antifungal effect of LAB CFS, the presence of organic acids was investigated by HPLC. The organic acid profile of the three bacteria is shown in Figure 2. A total of three organic acids were determined, namely acetic, lactic, and citric acids. The bacteria were grown in MRS broth, in which the only carbohydrate source is glucose, and produced lactic acid as the higher (*p* < 0.001) final product (19.1 to 24.0 g/L). Acetic acid was the second most abundant in all analyzed CFS, ranging from 8.6 to 10.5 g/L. The other organic acid quantified in the CFS was citric acid, which was only present in the CFS of *L. brevis* CCMA 1762 (2.1 g/L), probably derived from the MRS composition.

### 2.6. Screening of LAB Mycotoxin-Detoxifying Properties

**Figure 3:**
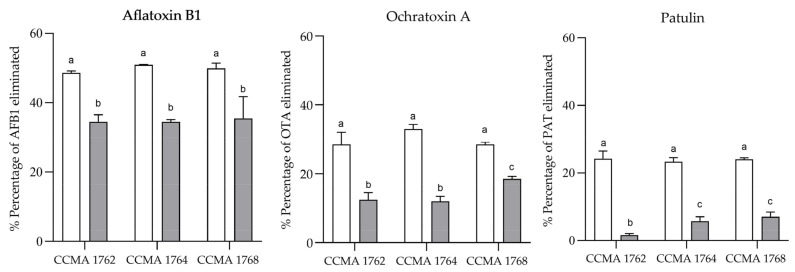
Percentage of AFB1, OTA, and PAT eliminated by the LAB culture in MRS-broth. Absorption (white), biotransformation (grey). The results are expressed as mean ± SD (*n* = 2). Different letters represent significant differences (*p* < 0.05) between strains within the same mycotoxin. Two-way ANOVA with Tukey’s multiple comparisons test.

The biotransformation and adsorption of AFB1, OTA, and PAT by the LAB strains after 12 days of cultivation in the MRS medium were studied to characterize further the anti-mycotoxins properties of selected LAB strains. The results obtained are depicted in Figure 3. The biotransformation experiments showed elimination percentages between 34.4% and 35.6% for AFB1, 12.1–18.2% for OTA, and 1.6–7% for PAT, depending on the LAB strain. On the other hand, the adsorption experiments showed higher percentages of mycotoxin elimination, ranging from 48.5% to 51% for AFB1, 28.5% to 33.1% for OTA, and 23.3% to 24.2% for PAT. For all bacteria tested, the elimination percentages of the adsorption experiments were significantly higher (*p* ≤ 0.01) than those of the biotransformation assays. Therefore, the tested LAB were generally more efficient at binding the mycotoxins to their cell walls than at biotransforming them. Moreover, the chromatograms of these assays did not show any relevant peak that could explain the biotransformation data obtained. Nonetheless, since the AFB1 disappearance (≈35%) was still considerable, we revised the existing literature and conducted further experiments to advance a possible biotransformation pathway that could explain this observation.

### 2.7. Evaluation of AFB1 Biotransformation into AFB2a

**Figure 4:**
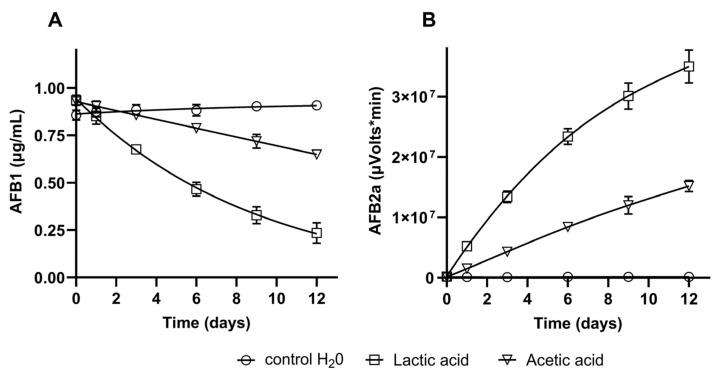
Biotransformation of AFB1 by lactic and acetic acid (0.1 M) during 12 days at 30 °C. (**A**) AFB1 concentration in assays, (**B**) AFB2a peak area in assays. Control: distilled water + AFB1 (1 µg/mL).

The two primary organic acids identified in the LAB supernatant (lactic and acetic acid) were tested to evaluate their effect on AFB1, and the results are shown in Figure 4. After an incubation time of 12 days, a reduction of AFB1 content of 73% was observed in the presence of lactic acid, whereas the reduction achieved with acetic acid was 27% (Figure 4A). The HPLC analysis also showed that AFB1 was biotransformed into AFB2a, as the peak area of this compound increased over time with both acids (Figure 4B). Further, the accumulation of AFB2a was more expressive in the presence of lactic acid (3.7 × 10^7^ µVolts/min) than with acetic acid (1.5 × 10^7^ µVolts/min), which agrees with AFB1 contents in the respective assays. An example of obtained chromatograms is presented in Figure S1. The left chromatograms depict samples collected at the incubation time 0, and the right chromatograms are from samples of day 12. The only relevant peak observed in the control samples (water) is AFB1, with a retention time of 6.1 min (Figure S1A,B). On the other hand, after 12 days, the samples containing lactic and acetic acid depict a second prominent peak at the retention time of 2.7 min, corresponding to AFB2a (Figure S1D,F). The same is observed in the chromatograms of MRS control (Figure S1G,H) and of strain *L. paracasei* CCMA 1764 cultivated in MRS (Figure S1I,J). After 12 days of incubation with the LAB, the peak of AFB2a appeared with an area of 5.3 × 10^6^ µVolts/min, and the AFB1 concentration in the assay was reduced 29% relative to the initial value of 10 µg/mL.

## 3. Discussion

There is considerable interest in microorganisms as biocontrol agents to reduce the concomitant proliferation of fungi and mycotoxins. Nonetheless, to select the most active strains, it is crucial to study both their ability to restrain fungal growth and their capacity to suppress the production of mycotoxins since there exists evidence that, in particular conditions, their production can be stimulated [28,29]. The fermentation of table olives is traditionally conducted by lactic acid bacteria and yeasts that progressively drop the brine pH and catalyze oleuropein into non-bittering compounds via their β-glucosidase and esterase enzymes [30]. Therefore, the microorganisms involved in this process can be a potential source of biocontrol agents once they proliferate in an extreme environment (up to 12% of salt and polyphenols), where molds often also manage to grow. In a previous study [14], we isolated LAB strains from Brazilian naturally fermented table olives, the antifungal and antimycotoxigenic properties of which are reported here.

The evaluation of LAB antifungal effects against *A. flavus*, *A. carbonarius*, *P. nordicum*, and *P. expansum* was first conducted by the dual-culture overlay method. This assay showed that tested LAB were more active against the *Penicillium* species than the *Aspergilli* and that strains *L. paracasei* subsp. *paracasei* CCMA 1764, *L. brevis* CCMA 1762, and *L. pentosus* CCMA 1768 possessed the strongest and broadest growth-inhibitory properties. Consequently, the cell-free culture supernatant of these strains was further characterized by the food poisoning method against *A. flavus*, *A. carbonarius*, and *P. expansum*. Their effect on fungal growth was evaluated by monitoring the colonies’ radial growth on plates and absorbance readings on microplates. Moreover, the produced mycotoxins were quantified in both assays and compared with controls. With this approach, the antifungal effect of LAB strains could be tested in a solid and liquid medium.

The data of plate and microplate methods agreed well when 500 µL/mL of CFS were used in the assays. However, when 300 µL/mL of CFS were used, the inhibitions were more heterogeneous. In that case, the microplate method gave higher inhibitions, particularly for *P. nordicum* MUM 08.16 (Table 2). The liquid medium may potentiate the inhibitory effects of LAB CFS once it allows better diffusion of the inhibitory compounds. In addition, the smaller volumes of culture medium used in this assay may also have influenced fungal growth, as low availability of nutrients can render fungi more susceptible to the inhibitors. Still, both methods permitted following the growth of fungi for seven days and to evaluate the antifungal strength of the LAB strains. Furthermore, in both assays, it was observed that growth inhibitions increased with increasing CFS concentrations. Russo et al. [31] reported a similar behavior with the CFS of *L. plantarum* strains against species from the *Penicillium*, *Aspergillus*, *Fusarium,* and *Cladosporium* genera.

In detail, the antifungal assays showed that *A. flavus* MUM 08.201 and *A. carbonarius* 01UAs293 were more resistant to LAB supernatants (particularly evident when 300 µL/mL of CFS was used). This result is in line with other studies in which the resistance of *Aspergillus* fungi to LAB inhibitory effects was more substantial compared to *Penicillium* species [14,32,33,34,35]. On the other hand, when 500 µL/mL were used, the CFS of strains *L. paracasei* CCMA 1764 and *L. pentosus* CCMA 1768 showed the strongest growth-suppressing effect on tested *Aspergilli*, which is an interesting result once most species of this genera are weakly sensitive to antifungal agents [36]. Concerning the *Penicillium*, all the analyzed supernatants almost completely inhibited the growth of tested species at a concentration of 500 µL/mL after seven days of incubation. On the other hand, when 300 µL/mL of CFS was used, it was observed that *P. expansum* MUM 17.86 was more sensitive to CFSs than *P. nordicum* once the inhibition percentages were almost two times higher. Chen et al. [37] also found *P. expansum* very sensitive to LAB, with its growth being entirely suppressed by the CFS of an *L. kefiri* strain. Moreover, the observed effect on *P. nordicum* MUM 08.16 was substantially higher than that of *L. plantarum* and *L. buchneri* strains studied by Guimarães et al. [13]. In the work of [38], the *P. verrucosum* was the most sensitive fungi to LAB treatments, while the least sensitive were fungi of the genus *Aspergillus*.

*Aspergillus* and *Penicillium* are the main fungal genera that cause deterioration problems in table olives [39]. Their development during olives fermentation leads to fruits’ softening and sensorial defects such as musty off-odors and taste, but it can also endanger consumers’ health if mycotoxins are involved [11]. Therefore, LAB isolated from table olives that show more vigorous inhibiting activity against these fungi are of interest as future starter cultures to improve the safety and quality of fermented olives. Other authors have tested the antifungal activity of bacteria isolated from fermented olives [14,40,41]. In their work, Abouloifa et al. [42] evaluated *L. plantarum, L. pentosus,* and *L. brevis* isolates, and all strains’ CFS showed antifungal activity against the mycelial growth of *A. niger* and *P. digitatum*, reaching percentages of inhibition between 23% and 33% and 27% and 40%, respectively. Panebianco and Caridi [41] also reported that 91% of the isolated LAB strains exhibited antagonistic activity against *P. crustosum* and 18% against *Aspergillus* sp., with the most representative strains being identified as *L. pentosus* and *Fructilactobacillus sanfranciscensis*. Simões et al. [14] and Riolo et al. [43] identified several LAB, isolated from olives, that showed strong inhibiting properties against *P. nordicum*. The potential of LAB as biopreservative agents in black olives was also studied in the work of El Oirdi et al. [44], in which CFS from *L. plantarum* showed an important bioprotective effect against attack by *A. fumigatus*, delaying fungus growth for about 30 days, pointing to the possible use of indigenous isolates of lactic acid bacteria in the prevention of foods, such as fermented table olives.

Considering the effect of CFS in the production of the mycotoxins, it was observed that the studied LAB were more efficient in hindering PAT production than AFB1 and OTA in both plate and microplate assays (Table 3). In addition, the mycotoxins results agreed with fungal growth inhibitions, suggesting that the mycotoxin reductions were primarily due to the reduction of fungal growth rather than resulting from interferences in their biosynthesis. This behavior was more evident with the fungus *P. expansum* because the decline of PAT correlated well with the high reductions of fungal growth. On the other hand, the reduction in AFB1 concentration (up to 31%) observed with 300 µL/mL of CFS demonstrates some antimycotoxigenic effect that is not dependent on mycelia growth because their inhibition in those conditions reached at most 18%. Similar effects were reported by Guimarães et al. [13] concerning an *L. plantarum* strain and several aflatoxigenic fungi. In the case of OTA, only the 500 µL/mL concentration could reduce its production (65–100%), but these values also depended on the considerable reduction of *A. carbonarius* growth, as for PAT.

According to Sadiq et al. [45], the organic acids produced by LAB are the main agents that efficiently affect fungal growth. Their lipophilic nature and protonated form at low pH facilitates their diffusion through the fungal membrane and causes damage to fungal cells because they accumulate in the dissociated form into the cytoplasm. Still, LAB can produce many metabolites that possess antifungal properties, and often the synergetic or additive effect of the multiple produced compounds are responsible for the strain’s antifungal potency. According to Siedler et al. [20], some of the involved inhibitory mechanisms include membrane destabilization, proton gradient interference, enzyme inhibition, and the creation of reactive oxygen species.

The main product of LAB carbohydrate metabolism is lactic acid, but other compounds may also be formed, depending on the fermentative properties of the species. For example, depending on the conditions, the heterofermentative species ferment sugars into lactic acid, acetic acid, CO2, and/or ethanol [46]. The most representative metabolites detected in the spontaneous fermentation of green and black olives are lactic and acetic acids, being also detected some citric, tartaric, and malic acids that came from olive fruits’ pulp [47,48,49,50,51]. In this work, lactic acid was the predominant compound found in LAB supernatants (Figure 2), which agrees with other studies [52,53]. Lactic acid was quantified in the range of 19.1–23.9 g/L in the supernatants, which is in line with Nazareth et al. [54], who detected 15 g/L of lactic acid in the CFS of an *L. plantarum* strain that exhibited antifungal activity. In addition, strains *L. brevis* CCMA 1762, *L. paracasei* CCMA 1764, and *L. pentosus* CCMA 1768 produced all acetic acid in the range of 8.6–10.5 g/L. The production of acetic acid by LAB strains can result from the degradation of produced lactic acid [55], from citrate metabolism [56], or the heterofermentative pathway [57]. Acetic acid exhibits potent inhibitory activity against fungi due to its higher pKa value [45]. However, lactic acid also possesses antifungal properties [58].

To complete the characterization of the isolates *L. brevis* CCMA 1762, *L. paracasei* CCMA 1764, and *L. pentosus* CCMA 1768, we studied their capacity to adsorb or biotransform AFB1, OTA, and PAT. The strains were cultivated in the presence of the mycotoxins, and the supernatants were analyzed after centrifuging or not the cells. Mycotoxin reductions of 23–51% were observed in the supernatants after the centrifugation step, suggesting that strains’ cells could remove the mycotoxins by a binding phenomenon. On average, the binding effect was more pronounced with AFB1 than with OTA and PAT. Several authors consider that the adsorption of mycotoxins is the primary mechanism responsible for LAB antimycotoxigenic effects [45,59,60]. Typically, the mycotoxins adhere to the peptidoglycan structure of LAB cell walls, reducing their bioavailability, and consequently their toxicity since it prevents their intestinal absorption. Nonetheless, this phenomenon is strain-specific once different strains of the same species may have different bind capacities for specific mycotoxins [61]. Several LAB have been reported to be capable of binding PAT [15,62], OTA [63], and AFB1 [63,64].

On the other hand, when the supernatants were extracted together with the cells, the mycotoxin reductions were between 2% and 36%. In particular, they were more expressive for AFB1 (34–36%) than for the other mycotoxins, suggesting that AFB1 could be biotransformed during the cultivation of LAB. To elucidate this finding, we conducted experiments with lactic and acetic acid solutions since there exist reports of the conversion of AFB1 into AFB2a in the presence of these and other organic acids when heated at high temperatures [65,66]. Figure 4 and Figure S1 show this conversion also occurs at lower temperatures (30 °C) when AFB1 was incubated with the two major organic acids produced by LAB, lactic, and acetic acid. AFB2a peak was clearly observed in chromatograms D and F of Figure S1. In addition, it was detected too in chromatogram J, which corresponds to the incubation of *L. paracasei* CCMA 1764 with 10 µg/mL of AFB1 for 12 days. AFB2a is a hemiacetal form of AFB1, whose mutagenic potency was estimated to be 1000 times inferior to that of AFB1 [67]. Therefore, we demonstrate that LAB can effectively detoxify this mycotoxin by this route. Nonetheless, this is a slow chemical process, which requires long incubation periods once the organic acids produced by the LAB seem to be the main agents involved.

The use of beneficial bacteria and other microorganisms for counteracting health problems associated with mycotoxins is a reality, with numerous research works, products, and patents being released during the last decades [68,69]. The underlying strategy consists of supplying microorganisms that adsorb or biotransform mycotoxins to ultimately exert their effects on the digestive tract of humans and animals. The utilization of LAB strains with improved or confirmed biotransformation and absorption capabilities is one of the determinant aspects of this approach. In this context, the fermentation process of table olives is a rich ecosystem that contains a complex community of bacteria and yeasts, with the edible olives being a source of valuable microorganisms that can positively modulate the intestinal microflora [70]. Therefore, it was crucial to investigate these LAB isolates for their anti-mycotoxin properties.

## 4. Conclusions

This work investigated the antifungal and anti-mycotoxins properties of some lactic acid bacteria isolated from naturally fermented Brazilian table olives. Among all the isolates, the strains *L. brevis* CCMA 1762, *L. paracasei* CCMA 1764, and *L. pentosus* CCMA 1768 showed the most important antifungal and anti-mycotoxins activities against tested fungal species. Furthermore, analyses of their cell-free supernatants have shown the production of organic acids, mainly lactic and acetic acids. In general, the properties of these bacteria make them a promising biological solution to control contamination by fungi of the genus *Aspergillus* and *Penicillium* and reduce mycotoxin levels in food or feed systems. In addition, the studied bacteria can be attractive as starter cultures to improve the safety of table olives fermentation, providing a natural solution to control toxigenic fungi during the process and improve the safety and quality of final products. By employing well-characterized lactic acid bacteria, the chances of successful fermentations increase, and detrimental changes in the fermenting microbial community and the physicochemical and sensory parameters of the fruits can be prevented. In addition, these table olives can be an edible source of LAB with anti-mycotoxin attributes, reinforcing the individuals’ intestinal microflora and increasing their resistance to mycotoxin-induced health problems.

## 5. Materials and Methods

### 5.1. Chemicals and Culture Media

De Man, Rogosa, and Sharpe (MRS) broth and agar were purchased from Oxoid (Hampshire, UK) and used for the growth of the LAB, while malt extract agar base with mycological peptone (MEA) supplied by Himedia (Mumbai, India) was used for all fungal cultures. Both MRS media were supplemented at 20% with Tomato Juice broth supplied by Fluka (Charlotte, NC, USA). Malt Soft Agar (MSA) supplied by Himedia (Mumbai, India) was used for the initial screening of LAB antifungal properties, consisting of malt extract (2.5%), bacteriological peptone (0.1%), glucose (2.5%), and bacteriological agar (1%) provided by Oxoid (Hampshire, UK). The malt extract broth (MEB) used in the microplate inhibition assays was purchased from Himedia (Mumbai, India). Peptone, Tween 80, and glycerol were supplied by Fisher Scientific (Porto Salvo, Portugal).

For the extraction of mycotoxins and preparation of the mobile phase, HPLC grade methanol (VWR, Carnaxide, Portugal), HPLC grade acetonitrile (Fisher Scientific, Porto Salvo, Portugal), and acetic acid (Fisher Scientific, Porto Salvo, Portugal) were used. For the analysis of organic acids, the reagents used were: DMSO (Dimethyl sulfoxide) (Merck, Darmstadt, Germany), ethyl acetate (Fisher Scientific, Porto Salvo, Portugal), formic acid (Sigma-Aldrich, Algés, Portugal), magnesium sulfate (LabKem, Barcelona, Spain), and sodium chloride (VWR, Carnaxide, Portugal). To prepare the organic acid, individual stock solutions of lactic acid from PanReac AppliChem (Darmstadt, Germany), and citric acid and acetic acid from VWR (Carnaxide, Portugal) were used. All mycotoxin standards were acquired from Sigma-Aldrich (Algés, Portugal). All analytes had a purity of >95%.

### 5.2. Lactic acid Bacteria Strains

A total of 14 LAB strains were studied (Table S1). Strains belonging to the species *Levilactobacillus brevis* (CCMA1762, CCMA1765, CCMA1766), *Lacticaseibacillus paracasei* subsp. *paracasei* (CCMA1763, CCMA1764, CCMA1767, CCMA1769, CCMA1770, CCMA1771, CCMA1772, CCMA1773, CCMA1774, CCMA1775) and *Lactiplantibacillus pentosus* (CCMA1768). They were previously isolated from naturally fermented table olives (Greek-style) of cultivars Grappolo 541 and Ascolano, harvested at the green stage at the Experimental Farm of EPAMIG (Minas Gerais Agricultural Research Company, Minas Gerais, Brazil) and deposited in the Culture Collection of Agricultural Microbiology (CCMA) of the Federal University of Lavras. CCMA is registered at the World Data Centre for Microorganisms (WDCM) as CCMA-UFLA under the number WDCM 1083. The isolates were identified by amplifying 16S rRNA using the primers 27F (5′-AGAGTTTGATCCTGGCTCAG-3′) and 1512-R (5′-GGCTACCTTGTTACGACT-3′) (Devereux and Willis 1995). The amplified PCR products were sent for sequencing to Macrogen USA (MD, USA). The strains were grown for 48 h at 37 °C in MRS broth for assays. All the strains were stocked at −80 °C using glycerol (20% *v*/*v*) as a cryoprotectant agent.

### 5.3. Fungal Strain and Inoculum Production

Fungal strains used in the present work were *Aspergillus flavus* MUM 08.201, *Aspergillus carbonarius* 01UAs293, *Penicillium nordicum* MUM 08.16, and *Penicillium expansum* MUM 17.86, which were obtained from the MUM Culture Collection (Micoteca of University of Minho, Braga, Portugal). Fungi were reactivated in MEA agar in the dark at 25 °C. After an incubation period of 7 days, the fungal spores were suspended in a solution of 0.1% peptone and 0.05% Tween 80. The spore concentration in the inoculum was determined with a Neubauer chamber. After counting the spores, the concentration was adjusted to 10^4^ or 10^6^ spores/mL. During the trials, all spore solutions were stored at −20 °C.

### 5.4. Screening of LAB for Antifungal Properties

The initial screening to check the antifungal activity of LAB strains was carried out using the “dual-culture overlay assay” [71]. Briefly, bacteria were inoculated as 2 cm long lines in MRS agar plates and incubated at 37 °C for 7 days, allowing well-grown lines of bacteria to form. Then, plates were overlaid with 10 mL of MSA previously inoculated with 10^4^ fungal spores/mL of the target fungi and incubated for 7 days at 25 °C. Overlaid plates were visually examined for mold growth inhibition zone around the LAB streaks, and these data were used for selecting LAB strains with potential antifungal activity. The results were classified as strong inhibition +++ (formation of strong inhibition zones around the LAB culture); inhibition ++ (formation of inhibition zones around the LAB culture); some inhibition + (LAB were not overlaid by the fungi), or absent inhibition − (LAB were completely overlaid by fungal growth). Finally, the LAB strains that inhibited all tested fungal strains were selected for further analysis.

### 5.5. Antifungal Properties of LAB Cell-Free Supernatants

The strains *L. paracasei* subsp. *paracasei* CCMA 1764, *L. brevis* CCMA 1762, and *L. pentosus* CCMA 1768 were reevaluated against the same fungal strains by plate and microplate growth inhibition assays using their cell-free supernatant (CFS) and the food poisoning method. First, the bacteria were cultivated in MRS broth for 48 h at 37 °C, and bacterial cells were removed by centrifugation (7000 RCF, 10 min, 4 °C). Then, the cell-free supernatant was filtered through a sterile syringe filter (0.2 μm, PES) and stored at 4°C until use. The antifungal and antimycotoxigenic activity was evaluated by the plate and microplate methods in order to compare results and verify the consistency of data. In addition, at the end of the incubation period, the mycotoxins AFB1 (from *A. flavus*), OTA (from *A. carbonarius*), and PAT (from *P. expansum*) were extracted from culture media to evaluate potential anti-mycotoxins effects. All experiments were conducted in duplicate.

#### 5.5.1. Plate Inhibition Analysis

The “poisoned food technique” was used for the plate inhibition analyses according to Guimarães et al. [13] with modifications. First, the CFS was added at concentrations of 300 and 500 µL/mL of MEA medium and poured into plates. The composition of MEA was adjusted accordingly to maintain the nutritional composition of culture media consistent for each concentration of supernatant used. Afterward, 10 μL of the fungi spore suspension (10^6^ spores/mL) were inoculated in the center of the plate. For the control experiment, the CFS was replaced by the MRS broth culture medium. Plates were incubated for 7 days at 25 °C in the dark. The diameters of the fungal colonies were measured daily. After 7 days of growth, mycotoxins were extracted using the methodology described by Guimarães et al. [13]. Briefly, the contents of the Petri dishes were cut and placed in falcon tubes, and 20 mL of acetonitrile/methanol solution (80/20, *v*/*v*) was added. Subsequently, the falcon tubes were mixed vigorously and stored in the dark at 25 °C overnight. The falcons’ content was filtered using syringe filters (0.2 μm, nylon) into amber vials and analyzed by HPLC.

#### 5.5.2. Microplate Inhibition Analysis

The antifungal activity of selected LAB was also evaluated in 96-well microplates, according to the growth inhibition test reported by Ruggirello et al. [35], with some modifications. The CFS was added at 300 and 500 µL/mL concentrations to MEB medium, and inoculation was done with 10 μL of spore suspension (10^6^ spores/mL). Microplates were incubated for 7 days at 25 °C, and the absorbance was measured daily at 600 nm using a microplate reader (Synergy HT, Biotek, Vermont, USA). Positive controls (PC) consisted of 10 μL of spore suspension (10^6^ spores/mL) inoculated in MEB medium without the addition of LAB CFS, and negative controls (NC) consisted of MEB medium supplemented with 300 and 500 µL/mL of sterile fresh MRS broth. Results were reported as optical density (OD_600nm_), and at the end of the incubation period, the growth inhibition for each fungus was calculated using the values found in control wells. All samples were analyzed in duplicate, and the final assay volume was 200 µL. The percentage of inhibition was calculated using the formula:

Microbial inhibition=[1−(Ac−NCPC)]×100 

where Ac represents the absorbance of the well with a CFS concentration, NC is the negative control, and PC is the absorbance of positive control.

Mycotoxins were evaluated by extracting the full content of the wells (200 µL) with 400 µL of acetonitrile/methanol solution (80/20, *v*/*v*) in an Eppendorf tube. After vortexing vigorously, the tubes were stored in the dark at 25 °C overnight. Subsequently, the mixtures were filtered with syringe filters (0.2 µm, nylon) and analyzed by HPLC.

### 5.6. HPLC Analysis of Cell-Free Supernatant

The organic acids were analyzed by HPLC (Jasco) with UV-Visible detection (Jasco 870-UV-visible). The chromatographic separation was performed on an Aminex HPX-87H column (300 × 4.6 mm, 8 μm). A 5 M H_2_SO_4_ solution was filtered and degassed to use as eluent. The flow rate was set to 0.6 mL/min at 60 °C, and 20 μL were injected. Detection was carried out at 210 nm. ChromPass software (Jasco Deutschland) was used to analyze the chromatographic data. For each organic acid, individual stock solutions were prepared by dissolving the compounds in distillate H_2_O, and seven-point calibration curves were prepared at concentrations of 0.1–2 mg/mL for acetic acid (AA), 1–20 mg/mL for lactic acid (LA), and 0.05–0.7 mg/mL for citric acid. Peak areas were used for quantification.

### 5.7. Assessment of LAB Anti-Mycotoxins Properties

The ability of LAB cultures to biotransform or adsorb mycotoxins was evaluated according to Taheur et al. [72] with some modifications. Stock standard solutions of AFB1, OTA, and PAT were prepared in acetonitrile at 2 mg/mL and stored at −20 °C until use. MRS broth was spiked with 2 μg/mL of the mycotoxins. For each strain, 10 mL of mycotoxin-contaminated MRS broth was inoculated in duplicate with 0.1 mL of the prepared inoculum of strains *L. paracasei* subsp. *paracasei* CCMA 1764, *L. brevis* CCMA 1762, and *L. pentosus* CCMA 1768. Two negative controls were also prepared using 0.1 mL of sterile MRS instead of the inoculum. The tubes were mixed and incubated anaerobically at 37 °C for 12 days. In order to assess biotransformation, 1 mL of the tubes’ content was mixed with 1 mL of acetonitrile/methanol solution (80/20, *v*/*v*), vortexed for 1 min, and left overnight at 25 °C in the dark. In order to assess adsorption, the tubes were centrifuged (7000 RCF/10 min, 4 °C) and then processed similarly. After vortexing for 1 min, all samples were filtered into clean vials using a syringe filter (0.2 μm, nylon) and preserved at −20 °C until HPLC analysis.

### 5.8. Mycotoxins Analysis

The mycotoxins AFB1 and OTA were quantified by high-performance liquid chromatography (HPLC) with fluorescence detection. The system included a pump Varian Prostar 210, an autosampler Varian Prostar 410, a fluorescence detector Jasco FP-920 and a column heater Jones Chromatography 7971 (30 °C), operated by the interface Varian 850-MIB and Galaxie chromatography data system. The analytical column was a C18 YMC-Pack ODS-AQ (250 × 4.6 mm, 5 μm) coupled with the respective pre-column [14].

For AFB1, the mobile phase (1.0 mL/min) was water/acetonitrile/methanol (3/1/1, *v*/*v*/*v*). AFB1 detection was conducted after post-column derivatization using a photochemical reactor (PHRED unit from Aura Industries, New York, NY, USA) using the excitation (ex.) and emission (em.) wavelengths of 365 nm and 435 nm, respectively [73]. The determination of OTA was performed according to Abrunhosa et al. [74]; the mobile phase (1.0 mL/min) was acetonitrile/water/acetic acid (99/99/2, *v*/*v*/*v*), and detection was performed at ex.: 333 nm and em.: 460 nm. In both cases, the injection volume was 50 μL. The patulin mycotoxin was analyzed according to Wang et al. [75] with some modifications, using ultra-high-pressure liquid chromatography (UHPLC) on a Shimadzu Nexera X2 system equipped with SPD-M20A DAD detector and using a Brisa LC2 C18 Teknokroma column (250 × 4.6 mm, 5 μm). The mobile phase (0.75 mL/min) was water/acetonitrile (95/5, *v*/*v*), the detection was set to 276 nm, the temperature to 25 °C, and the injection volume to 20 μL. All mobile phases used went through filtration and degassing (0.2-μm membrane filter, GHP-Gelman). The retention times of AFB1, OTA, and PAT were approximately 20.3 min, 13.5 min, and 19.7 min, respectively.

For the quantification of mycotoxins, standard curves were prepared in acetonitrile. The standard concentration ranged from 0.005 to 1.0 μg/mL. Signal-to-noise was used to calculate the limit of detection (LOD) (3:1 ratio) and the limit of quantification (LOQ) (10:1 ratio). The obtained values for AFB1 were LOD = 0.004 μg/mL and LOQ = 0.013 μg/mL; for OTA, LOD = 0.005 μg/mL and LOQ = 0.017 μg/mL; and for PAT, LOD = 0.12 μg/mL and LOQ = 0.35 μg/mL. Mean recoveries were 93% ± 7.0 for AFB1, 113% ± 6.7 for OTA, and 97% ± 6.0 for PAT.

### 5.9. Evaluation of AFB1 Biotransformation into AFB2a

Tubes containing 20 mL of Milli-Q water with 0.1 M of lactic and acetic acid were sterile filtered with syringe filters (0.2 μm, PES) and spiked with 1 μg/mL of AFB1 by adding the appropriate amount of stock standard. Negative controls were prepared in the same way but without the organic acids. The mixtures were incubated in the dark at 30 °C without shaking over 12 days. Strain *L. paracasei* subsp. *paracasei* CCMA 1764 was cultivated as previously described in MRS broth spiked with 10 μg/mL of AFB1. MRS broth was used as control. Periodically, 0.8 mL of the tubes’ content was collected, added to the same amount of acetonitrile/methanol solution (80/20, *v*/*v*), and strongly vortex mixing for 1 min. After that, samples were filtered into clean 2-mL vials using syringe filters (0.2 μm, nylon) and preserved at −20 °C until HPLC analysis. Experiments were conducted in duplicate. In this case, AFB1 and AFB2a were determined using a UHPLC SHIMADZU Nexera X2 system, equipped with diode-array and fluorescence detectors, and a Synergi Hydro-RP Column (100 × 3 mm, 2.5 µm). The mobile phase was water/methanol/acetonitrile (6/3/1, *v*/*v*/*v*); the flow was set to 0.7 mL/min, and the temperature to 35 ºC. The analyses were performed without derivatization at an excitation wavelength (ex) of 365 nm and emission (em) of 450 nm. A 10 µL sample was injected.

### 5.10. Statistical Analysis

Statistical analyses were performed using GraphPad Prism version 8.01 for Windows (La Jolla, CA, USA). The ANOVA and the Scott–Knott test on tables and two-way ANOVA and Tukey’s multiple comparisons test on figures were performed to test significant differences reported as: **** for *p* ≤ 0.0001; *** for *p* ≤ 0.001; ** for *p* ≤ 0.01; and * for *p* ≤ 0.05. Results were expressed as mean values ± standard deviation (*n* = 2).

## Figures and Tables

**Table 1:** Screening of LAB strains antifungal activity.

Species	Antifungal Activity			
	*A. flavus* MUM 08.201	*A. carbonarius* 01UAs293	*P. nordicum* MUM 08.16	*P. expansum* MUM 17.86
*L. brevis* CCMA 1766	−	−	+++	+
*L. paracasei* CCMA 1763	+	++	++	+
*L. paracasei* CCMA 1772	−	−	+	++
***L. pentosus* CCMA 1768**	**+**	**++**	**+++**	**+++**
*L. paracasei* CCMA1775	++	−	+++	++
*L. paracasei* CCMA 1769	−	−	+	+
*L. paracasei* CCMA 1770	−	−	+	+
***L. brevis* CCMA 1762**	**+**	**+++**	**++**	**+++**
***L. paracasei* CCMA 1764**	**+**	**+++**	**+++**	**+++**
*L. paracasei* CCMA 1771	−	−	+++	++
*L. brevis* CCMA 1765	−	−	+++	+
*L. paracasei* CCMA 1767	−	−	+++	+
*L. paracasei* CCMA 1773	−	−	−	−
*L. paracasei* CCMA 1774	−	−	+	+

Antifungal activity after 7 days of fungal growth. Strong inhibition +++ (formation of strong inhibition zones around the LAB culture); inhibition ++ (formation of inhibition zones around the LAB culture); some inhibition + (LAB were not overlaid by the fungi); absence of inhibition − (LAB were completely overlaid by fungal growth). Highlighted at bold the most active LAB strains.

**Table 2:** Fungal growth inhibition (%) of LAB CFS tested by the plate and microplate assays.

	LAB Strains	Plate Method			Microplate Method		
Fungi		CCMA 1762	CCMA 1764	CCMA 1768	CCMA 1762	CCMA 1764	CCMA 1768
*A.* carbonarius 01UAs293	**CFS (300 µL/mL)**	0	0	0	12 ^b^ ± 1.2	12 ^b^ ± 1.4	27 ^c^* ± 2.3
*A. flavus* MUM 08.201		4.7 ^c^ ± 1.2	6.6 ^c^ ± 0.6	3.5 ^c^ ± 1.1	18 ^b^ ± 0.9	15 ^b^ ± 0.8	14 ^d^ ± 1.7
*P. nordicum* MUM 08.16		39 ^b^ ± 1.8	37 ^b^ ± 1.5	40.7 ^b^ ± 0.0	16 ^b^* ± 0.4	95 ^a^ ± 1.5	95 ^a^ ± 1.1
*P. expansum* MUM 17.86		78 ^a^* ± 1.1	92 ^a^ ± 1.1	92 ^a^ ± 0.9	87 ^a^ ± 2.1	93 ^a^* ± 0.9	89 ^b^ ± 2.4
*A. carbonarius* 01UAs293	**CFS (500 µL/mL)**	40 ^c^* ± 1.7	100 ^a^	100 ^a^	30 ^c^* ± 2.5	100 ^a^	100 ^a^
*A. flavus* MUM 08.201		78 ^b^* ± 0.6	100 ^a^	98 ^a^ ± 0.6	73 ^b^* ± 3.2	99 ^a^ ± 0.1	98 ^a^ ± 1.2
*P. nordicum* MUM 08.16		100 ^a^	100 ^a^	100 ^a^	100 ^a^	100 ^a^	100 ^a^
*P. expansum* MUM 17.86		77 ^b^* ± 1.2	100 ^a^	100 ^a^	96 ^a^ ± 3.0	100 ^a^	100 ^a^

Results are expressed as mean (%) ± SD, determined in duplicate. Mean values with different letters in columns (within same LAB CFS concentration) and * in rows (within same method) differ significantly (*p* < 0.05) by the Scott-Knott test.

**Table 3:** Mycotoxins concentration (µg/mL) and respective reductions (%) detected in plate and microplate assays.

Mycotoxins	LAB Strains		Plate Method		Microplate Method	
			Concentration (µg/mL)	Reduction (%)	Concentration (µg/mL)	Reduction (%)
AFB1	CCMA 1762	CFS concentration 300 µL/mL	4.21 ^a^ ± 0.7	18%	3.77 ^a^ ± 0.6	8%
	CCMA 1764		3.63 ^a^ ± 0.1	29%	2.85 ^a^ ± 1.0	31%
	CCMA 1768		3.79 ^a^ ± 0.2	26%	3.27 ^a^ ± 1.1	20%
	Control		5.13 ^a^ ± 0.4		4.11 ^a^ ± 0.8	
OTA	CCMA 1762		9.74 ^a^ ± 0.1	-	5.76 ^b^ ± 1.0	-
	CCMA 1764		8.49 ^a^ ± 1.7	-	3.83 ^b^ ± 0.7	-
	CCMA 1768		5.31 ^a^* ± 0.9	-	4.21 ^a^ ± 0.2	-
	Control		5.26 ^a^* ± 0.9		1.45 ^b^* ± 1.1	
PAT	CCMA 1762		7.71 ^a^ ± 0.9	89%	3.60 ^b^ ± 0.37	95%
	CCMA 1764		1.65 ^a^ ± 1.2	98%	0	100%
	CCMA 1768		1.38 ^a^ ± 1.46	98%	0	100%
	Control		73.62 ^a^* ± 6.0		69.71 ^b^* ± 0.5	
AFB1	CCMA 1762	CFS concentration 500 µL/mL	0.24 ^a^ ± 0	95%	0.06 ^a^ ± 0	98%
	CCMA 1764		0	100%	0	100%
	CCMA 1768		0	100%	0	100%
	Control		5.13 ^a^* ± 0.4		4.11 ^a^* ± 0.8	
OTA	CCMA 1762		1.81 ^a^ ± 1.3	65%	0.28 ^b^ ± 0.1	81%
	CCMA 1764		0	100%	0	100%
	CCMA 1768		0	100%	0	100%
	Control		5.26 ^a^* ± 0.9		1.45 ^b^ ± 1.1	
PAT	CCMA 1762		5.12 ^a^ ± 0.1	93%	3.27 ^a^ ± 0.2	95%
	CCMA 1764		0	100%	0	100%
	CCMA 1768		0	100%	0	100%
	Control		73.62 ^a^* ± 6.0		69.71 ^b^* ± 0.5	

Results are expressed as mean concentration (µg/mL) ± SD determined in duplicate and percentages (%). Aflatoxin B1 (AFB1), ochratoxin A (OTA), patulin (PAT). Mean values with different letters in rows (different methods) and * in columns (different LAB CFS within same mycotoxin) differ significantly (*p* < 0.05) by the Scott-Knott test. (-) There is no reduction of mycotoxins.

## Data Availability

Not applicable.

## Supplementary Materials

The following supporting information can be downloaded at: https://www.mdpi.com/article/10.3390/toxins15010071/s1, Table S1: Lactic acid bacteria isolated from table olives used in this study (*n* = 14); Figure S1: Example of obtained chromatograms of AFB1 biotransformation into AFB2a. (A) water sample—day 0; (B) water sample—day 12; (C) 0.1 M lactic acid—day 0; (D) 0.1 M lactic acid—day 12; (E) 0.1 M acetic acid—day 0; (F) 0.1 M acetic acid—day 12; (G) MRS-broth—day 0; (H) MRS-broth—day 12; (I) LAB CCMA1764 in MRS-broth—day 0; (J) LAB CCMA1764 in MRS-broth—day 12.
