# Supplementary material for: Brazilian Table Olives: A Source of Lactic Acid Bacteria with Antimycotoxigenic and Antifungal Activity
Source: Toxins (Basel). 2023 Jan 13;15(1):71. doi: 10.3390/toxins15010071 (PMC9866039; doi:10.3390/toxins15010071)
Supplement: Supplementary file 1 [file toxins-15-00071-s001.zip › toxins-2125155-supplementary.pdf]

toxins

Supplement

Brazilian Table Olives: A Source of Lactic Acid Bacteria with Antimycotoxigenic and Antifungal Activity

Luara Simões [1,2], Natália Fernandes [1,3], José Teixeira [4,5], Luís Abrunhosa [4,5,*] and Disney Ribeiro Dias [6,*]

1 Biology Department, Federal University of Lavras, Lavras 37200-900, Brazil
2 Centre of Molecular and Environmental Biology, University of Minho, 4710-057 Braga, Portugal
3 Chemistry Department, University of California, 95616 Davis, CA, USA
4 CEB—Centre of Biological Engineering, University of Minho, 4710-057 Braga, Portugal
5 LABBELS—Associate Laboratory, Braga/Guimarães, Portugal
6 Department of Food Science, Federal University of Lavras, Lavras 37200-900, Brazil
* Correspondence: d3024@deb.uminho.pt (L.A.); diasdr@ufla.br (D.R.D.);
Tel.: +351-253-601-986 (L.A.); +55(35)3829-5256 (D.R.D.)

Table S1. Lactic acid bacteria isolated from table olives used in this study (n = 14).

Species	N° of isolates	Code	Isolation: cultivar and fermentation time
Levilactobacillus brevis	3	CCMA1762	Ascolano (time 120 days)
		CCMA1765	Ascolano (fresh fruit)
		CCMA1766	Ascolano (time 60 days)
Lacticaseibacillus paracasei subsp. *paracasei*	10	CCMA1763	Ascolano (time 120 days)
		CCMA1764	Ascolano (time 60 days)
		CCMA1767	Ascolano (time 30 days)
		CCMA1769	Ascolano (time 120 days)
		CCMA1770	Grappolo (time 60 days)
		CCMA1771	Grappolo (fresh fruit)
		CCMA1772	Ascolano (time 60 days)
		CCMA1773	Ascolano (fresh fruit)
		CCMA1774	Grappolo (time 120 days)
		CCMA1775	Grappolo (time 30 days)
Lactiplantibacillus pentosus	1	CCMA1768	Ascolano (time 120 days)

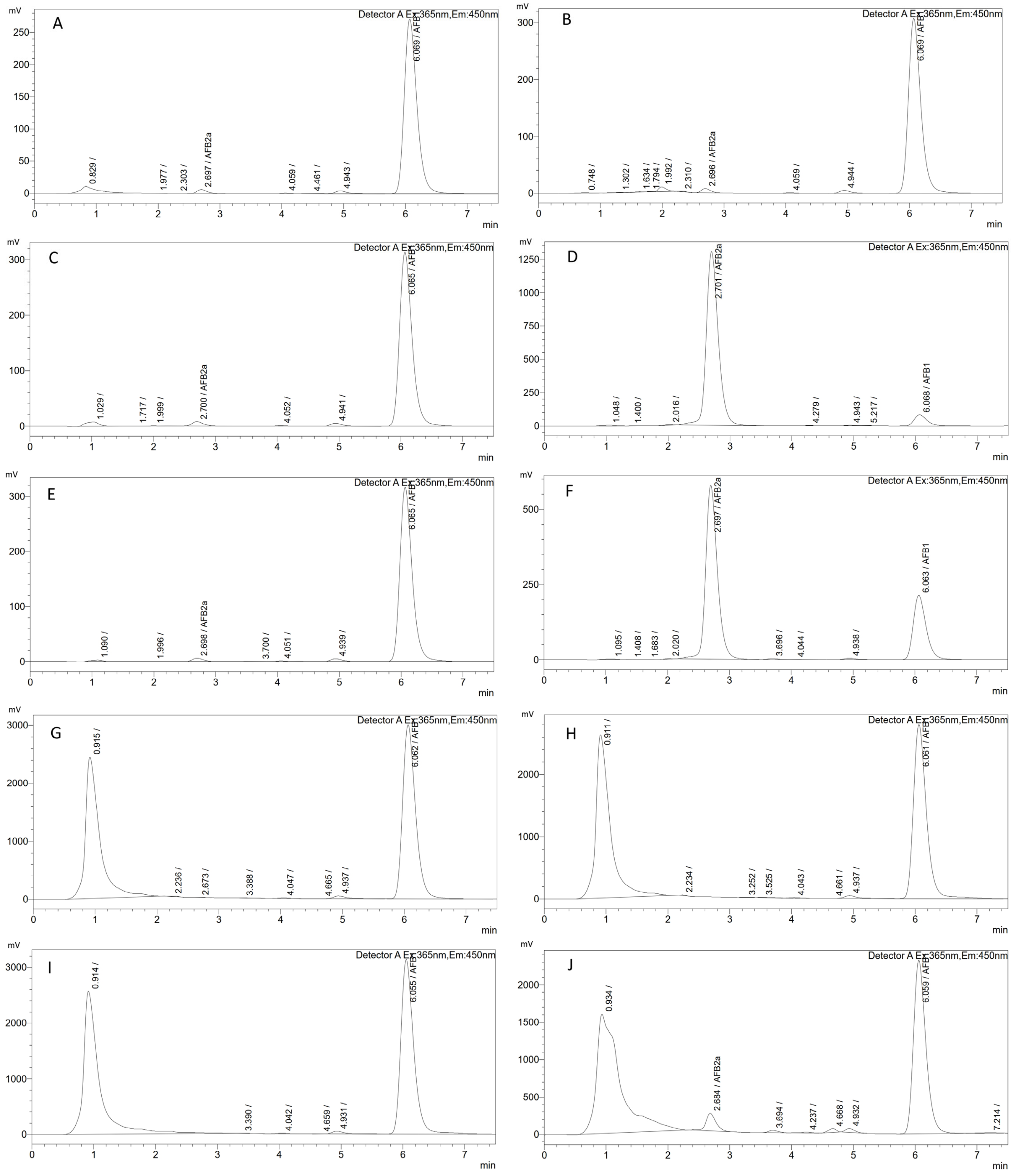

Figure S1. Example of obtained chromatograms of AFB1 biotransformation into AFB2a. (**A**) water sample – day 0; (**B**) water sample – day 12; (**C**) 0.1 M lactic acid – day 0; (**D**) 0.1 M lactic acid – day 12; (**E**) 0.1 M acetic acid – day 0; (**F**) 0.1 M acetic acid – day 12; (**G**) MRS-broth – day 0; (**H**) MRS-broth – day 12; (**I**) LAB CCMA1764 in MRS-broth – day 0; (**J**) LAB CCMA1764 in MRS-broth – day 12.
